# Single-Center Cross-Sectional Analysis of Patients with RA, SpA, and PsA: Data from the Prescription Database

**DOI:** 10.3390/jpm15080366

**Published:** 2025-08-11

**Authors:** Maurizio Benucci, Francesca Li Gobbi, Emanuele Antonio Maria Cassarà, Anna Lucia Marigliano, Alessandro Mannoni, Enrico Benvenuti

**Affiliations:** 1 Rheumatology Unit, S. Giovanni di Dio Hospital, USL-Toscana-Centro, 50143 Florence, Italy; francesca.ligobbi@uslcentro.toscana.it (F.L.G.); emanueleantonio.cassara@uslcentro.toscana.it (E.A.M.C.); 2 Pharmaceutical Governance and Appropriateness of Prescription Unit, Hospital S. Stefano, USL-Toscana-Centro, 59100 Prato, Italy; annalucia.marigliano@uslcentro.toscana.it; 3 Rheumatology Unit, P. Palagi Hospital, USL-Toscana-Centro, 50122 Florence, Italy; alessandro.mannoni@uslcentro.toscana.it; 4 Multidimensional Medicine Department, USL-Toscana Centro, 50143 Florence, Italy; enrico.benvenuti@uslcentro.toscana.it

**Keywords:** rheumatoid arthritis, psoriatic arthritis, spondilarthritis, prescription appropriateness

## Abstract

**Introduction**. The Italian Committee for Tailored BIOlogic Therapy (ITABIO), in a first report, has reviewed the literature to identify the best strategy for the choice of second-line biologic therapy in patients with rheumatoid arthritis (RA), spondyloarthritis (SpA), and psoriatic arthritis (PsA). To verify the application of ITABIO recommendations in real life and how the recommendations perform in maintaining the health status of patients affected by inflammatory arthritis (RA, SpA, PsA), a database has been developed by Pharmaceutical Governance to evaluate the appropriateness of prescriptions. **Methods**. We have analyzed retrospectively 616 patients, 288 (46.7%) affected by RA, 117 (19%) affected by SpA, and 211 (34.3%) affected by PsA. Age, sex, diagnosis, current treatment, previous treatments with csDMARDs, b-DMARDs, ts-DMARDs, presence of risk factors for cardiovascular (CV) events, liver disease, infections, extra-articular manifestations such as interstitial lung disease (ILD) for RA, enthesitis, dactylitis, uveitis, inflammatory bowel disease for SpA and PsA, neoplasms, diabetes, presence or absence of rheumatoid factor (RF) and anti-citrullinated peptide antibodies (ACPA) for RA were evaluated. **Results**. The percentage of treatments with anti-TNF biosimilars was 65.1, 52.4, and 24.3% in SpA (76 patients(pt)), PsA (110 pt), and RA (69 pt), respectively. The percentage of monotherapy was 68% (418 pt) in the three diseases. For RA, 34.2% of patients were difficult to treat (D2T) (98 pt), 54.8% (157 pt) were in monotherapy (tocilizumab-sarilumab-upadacitinib-filgotinib). Abatacept was the most prescribed treatment in RF and ACPA-positive patients and in those with ILD. The anti-IL-17A secukinumab was prescribed in 12% of SpA, of which 71% had enthesitis and dactylitis (14 pt). Ixekizumab was prescribed in 10.4% of PsA patients over 65 years with previous CV events, enthesitis, and dactylitis (21 pt). Apremilast was present in 71% of PsA with previous cancer. **Conclusions**. The cross-sectional analysis of prescriptions in patients with RA, SpA, and PsA demonstrates how the ITABIO recommendations can guide towards the correct appropriateness of prescription. RA and especially D2T-RA remains the disease with the greatest therapeutic failures, with the highest percentage of monotherapy (anti-IL-6 and Jak-i) and of discontinuation of MTX.

## 1. Introduction

In the EULAR recommendations for rheumatoid arthritis (RA) the working group agreed on five general principles and eleven recommendations regarding the use of conventional synthetic (cs) DMARDs (methotrexate (MTX), leflunomide, sulfasalazine) glucocorticoids (GC), biological (b) DMARDs (tumor necrosis factor inhibitors (TNFi) such as adalimumab, certolizumab pegol, etanercept, golimumab, infliximab including biosimilars), abatacept, rituximab, tocilizumab, sarilumab and targeted synthetic (ts) DMARDs (Janus kinase inhibitors such as tofacitinib, baricitinib, filgotinib, upadacitinib). Guidance is given for monotherapy, combination therapy, treatment strategies (treat-to-target), and tapering in sustained clinical remission. Safety aspects, including the risk of cardiovascular (CV) events (MACE: major adverse cardiovascular events) and malignancies, have been taken into account, together with costs and sequencing of b/tsDMARDs. Initially, MTX plus GC is recommended and in case of insufficient response within 3–6 months, treatment should be based on stratification according to risk factors: with poor prognostic factors (presence of autoantibodies, high disease activity, early erosions or failure of two csDMARDs), any bDMARD should be added to the csDMARD; after careful assessment of the risks of MACE, malignancies and/or thromboembolic events, tsDMARDs can also be considered at this stage. If the first bDMARD (or tsDMARD) fails, any other bDMARD (or another of the same class) or tsDMARD (considering the risks) is recommended [1]. Five general principles and 15 recommendations focusing on personalized medicine have been agreed upon for spondyloarthritis (SpA). The first five recommendations focus on the goal of treatment and on the monitoring of non-pharmacological management and of non-steroidal anti-inflammatory drugs (NSAIDs) as first-line treatment. Recommendations 6–8 address the use of analgesics and advise against long-term glucocorticoids and DMARDs utilization for pure axial involvement. Recommendation 9 describes the indication of bDMARDs (TNFi), interleukin-17 inhibitors (IL-17i), and tsDMARDs (Janus kinase inhibitors (Jak-i)) for patients with SpA with a disease activity score ≥ 2.1 and failure of ≥2 NSAIDs and with elevated C-reactive protein, sacroiliac joint inflammation by MRI, or radiographic sacroiliitis. Current practice is starting with a TNFi or IL-17i. Recommendation 10 addresses extra-musculoskeletal manifestations with anti-TNF monoclonal antibodies preferred for recurrent uveitis or inflammatory bowel disease, and IL-17i for significant psoriasis. In recommendation 11, the treatment failure should prompt the reassessment of diagnosis and consideration of comorbidities. In recommendation 12, if active axSpA is confirmed, switching to another b/tsDMARD is recommended. In recommendation 13, in patients with prolonged remission, gradual reduction rather than immediate discontinuation of bDMARD may be considered [2]. The updated recommendations for PsA include seven general principles and eleven recommendations and provide a treatment strategy for drug therapies. NSAIDs should be used as monotherapy only for mild and short-term PsA; oral GCs are not recommended. In patients with peripheral arthritis, early initiation of DMARDs is recommended, and MTX is preferred. If the treatment goal is not achieved with this strategy, a bDMARD should be started, without preference between modes of action. Severe cutaneous psoriasis should be addressed with bDMARDs targeting inhibitors of interleukin (IL)-23p40, IL-23p19, IL-17A, and IL-17A/F. In case of predominant axial or entheseal disease, an algorithm is also proposed. The use of Jak-i is mainly suggested after bDMARDs failure, taking into account relevant risk factors, or if bDMARDs are not the appropriate choice. Inflammatory bowel disease and uveitis, if present, should influence drug choices, with monoclonal TNFi [3]. The EULAR recommendations, described thus far, place second-line therapies on the same level and do not provide a guide on the criteria of choice, with some exceptions. The Italian Committee for Tailored BIOlogic Therapy (ITABIO), in a first report, has reviewed the literature to indicate the best strategy for the second-line choice of biologic therapy in patients with RA, SpA, and PsA. Among the variables analyzed, patient preference, the indication for TNFi monotherapy in women of childbearing potential, and the intravenous route with dose titration in obese subjects were found to be valid for all three rheumatic conditions. In RA, inadequate response secondary to etanercept (ETN) should be treated with a biologic agent other than TNFi, and after two or more anti-TNF failures, it is recommended to switch to a drug with a different mode of action, such as abatacept (ABA), rituximab (RTX), and tocilizumab (TCZ). In patients with PsA and SpA who fail the first TNFi, a switch to a second TNFi strategy is recommended, taking into account the evidence of efficacy of adalimumab (ADA) in patients with uveitis and intestinal manifestations. The severity of psoriasis, joint involvement, and the predominance of enthesitis and/or dactylitis may guide the choice towards anti-IL-23 or anti-IL-17 [4]. In a subsequent update on RA, several clinical variables related to specific drugs and host characteristics were analyzed to guide the choice towards anti-TNF b-DMARDs, non-anti-TNF b-DMARDs, or tS-DMARDs, thus allowing for personalized therapy, taking into account the results of the literature when the chosen variables predominated over the economic aspect [5]. Despite EULAR recommendations and the “treat to target” strategy in our clinical practice, we must take into account payer recommendations, and policy guidelines suggest a therapeutic choice toward the least costly treatment for the National Health System. To verify the real-world application of ITABIO recommendations and their effectiveness in maintaining the health of patients with arthritis (RA, SpA, PsA), the Pharmaceutical Governance has developed a database to assess the appropriateness of prescriptions.

## 2. Materials and Methods

To monitor the treatment of patients with arthritis (RA, SpA, PsA), at the S. Giovanni di Dio Hospital in Florence, the Rheumatology Unit team, in collaboration with hospital pharmacists, prepared a user-friendly database to record prescriptions for the last 5 years (January 2020/December 2024). The following factors that may influence the therapeutic choice have been evaluated: age, sex, diagnosis, ongoing treatment, previous treatments with cs-DMARD, b-DMARD, ts-DMARD, presence of risk factors for CV events (ASCVD calculator), liver disease, infections, extra-articular manifestations such as interstitial lung disease (ILD) for RA, enthesitis, dactylitis, uveitis, inflammatory bowel disease for SpA and PsA, neoplasms, diabetes, presence or absence of rheumatoid factor (RF), and anti-citrullinated peptide antibodies (ACPA) for RA. We have analyzed retrospectively these data to identify the different use of the same drugs in the three types of arthritis and to evaluate whether the presence of risk factors can guide the therapeutic choice. The observational study was reviewed and approved by the GISEA Project Ethics Review Board on 22 September 2020 (Code of Ethics s6496_OSS). Written informed consent was not required for participation in this study in accordance with national legislation and institutional requirements.

### Statistical Analysis

Since the data have a Gaussian distribution, we applied descriptive statistics, utilizing average and standard deviation (SD). The *t*-test for independent samples was used to check statistical differences for the different treatments. To compare the incidences, expressed by percentage (%), we used Fisher’s exact test. Statistical analysis was performed by © 2024 MedCalc Software Ltd. (v22.021, Acacialaan 22, 8400 Ostend, Belgium). The multivariate analysis has been conducted by INSTAT, GraphPad Software, version 3.10, last version 13 September 2023.

## 3. Results

The population analyzed retrospectively is made up of 616 patients, 288 (46.7%) affected by RA, 117 (19%) affected by SpA, and 211 (34.3%) affected by PsA.

**Figure 1:**
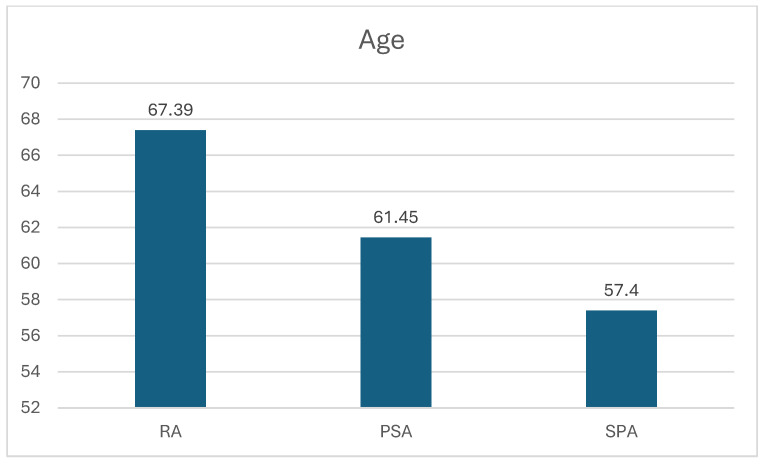
Average age in the three diseases.

Comparing the average age, patients affected by RA are the oldest, 67.39 ± 14.83 years old, followed by PSA patients, 61.45 ± 13.23 years old. The youngest are patients affected by SpA, 57.54 ± 13.06 years old (RA vs. PSA *p* = 0.0001; RA vs. SpA *p* = 0.0001; PsA vs. SpA *p* = 0.01) (Figure 1).

**Figure 2:**
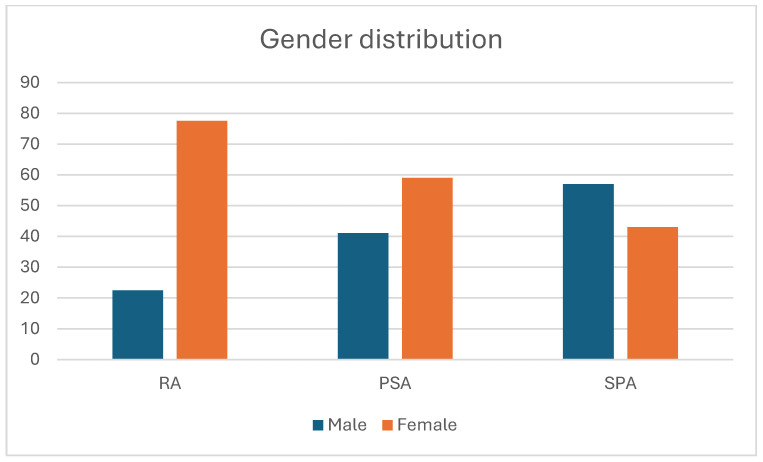
Gender distribution in the three diseases analyzed.

About the gender distribution, in the RA population, females are 77.5% and males 22.5% of patients, in the SpA group, males are 57% and females 43%, and in the PsA population, females are 59% and males 41%. Comparing the groups, there is a statistically significant difference in the number of females in RA versus PsA (*p* = 0.002) and SpA (*p* = 0.00008), and there is no difference between PsA and SpA. If we consider the male population, there is a statistically significant difference between RA and SpA (*p* = 0.0019). However, there is no statistically significant difference between RA and PsA and between PsA and SpA (Figure 2).

**Figure 3:**
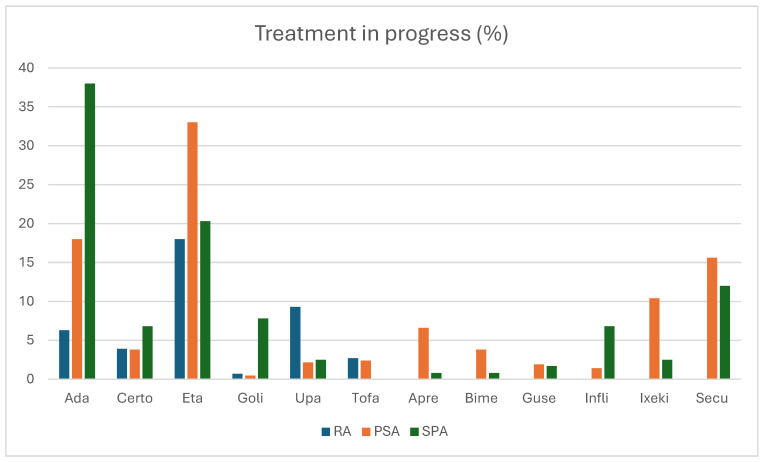
Distribution of treatments with drugs with multiple indications.

It is very interesting to analyze the distribution of treatment in relation to drugs with multiple indications. As shown in Figure 3, ADA is used to a lesser extent in RA patients compared to PsA and SpA patients, and it is also used less frequently in PsA patients compared to SpA patients. Patients with RA are treated with ETN less than patients with SpA and PsA. About golimumab (GOL), the percentage of RA patients treated with this drug is less than that of SpA patients, and the percentage of PsA patients treated with GOL is less than that of SpA patients. Upadacitinib (UPA) is used mainly in RA with a statistically significative difference compared with SpA and PsA. There are no differences in percentages of patients treated with tofacitinib (TOF) in RA and PsA. Apremilast (APR) is used mainly in PsA compared with SpA, as well as ixekizumab (IXE), while the opposite result is found with infliximab (INF). No differences in the percentage of patients treated in the three groups of arthritis are detected for bimekizumab (BIM), guselkumab (GUS), and secukinumab (SEC) (Table 1).

Another characteristic of the therapeutic choice for the three diseases is represented by the percentage of patients managed with monotherapy. Evaluating the data, it is possible to observe that in RA, 68% of patients are treated with a single advanced therapy, as well as 87% in PsA and 85% in SpA, but the differences are not statistically significative. When we have to choose a specific drug, it is important to consider the CV risk; therefore, we decided to evaluate the percentage of patients with this risk factor in the three different diseases. The result is that 44% of RA patients have a risk for CV events, 34% of PsA patients, and 27% of SpA patients. Comparing these data, there is a statistically significative difference only between RA and SpA (*p* = 0.034). We also decided to perform a multivariate analysis to identify any interactions between variables that the univariate analysis does not allow. This process allows us to isolate the actual effect of each variable in a more complex system, which is therefore closer to reality. The limit of this kind of approach is that the data comes from a database that records prescriptions and therefore lacks many clinical variables. Therefore, the result will be closely linked to the characteristics considered, which could be influenced by elements not present in the database. In a multiple regression, we analyzed the impact of the variable in the different group of patients treated with different drugs in RA (sex, age, cardiovascular risk, hepatotoxicity, infection, antibodies, pulmonary fibrosis, neoplasia and diabetes), SpA and PsA (sex, age, cardiovascular risk, hepatotoxicity, infection, antibodies, pulmonary fibrosis, neoplasia, diabetes, vasculitis, dactylitis and gut involvement). The t-ratio values relative to the variables considered are all below the significance threshold (<2), and all the *p*-values are over 0.05. By another multiple regression, we analyzed the patients treated with each single drug. The results show that the variables are independent of each other, as confirmed by the multicollinearity test, where all the R-squared results are <0.75. In conclusion, none of the variables considered seem to influence the choice of therapy.

### 3.1. Rheumatoid Arthritis Patients

To understand if there are some features that can guide the choice of the treatment, we have analyzed in RA patients the characteristics reported in Table 2.

About the age, the statistically significant differences are in ABA vs. CER group *p* = 0.0019, ABA vs. TOF *p* = 0.0006, ABA vs. UPA group *p* = 0.0033, showing a population older than the other one treated with different drugs. CER seems to be chosen in the youngest population, and the differences are statistically significant compared with ETA *p* = 0.009, FIL *p* = 0.0056, SAR *p* = 0.0023, TOC *p* = 0.0005, and UPA *p* = 0.0094. Patients treated with ETA are younger than those treated with TOF *p* = 0.0148; patients treated with FIL are older than patients treated with TOF *p* = 0.0085; patients treated with SAR are older than those treated with TOF *p* = 0.0025 and with UPA *p* = 0.023. Patients in the TOC group are older than patients in the TOF group *p* = 0.0014 and UPA group *p* = 0.014 and patients treated with TOF are younger than patients treated with UPA.

About the concomitant treatment, ABA and ADA have the greatest percentage of patients treated in combination therapy with csDMARD. (ABA vs. BAR *p*= 0.000019; ABA vs. ADA *p* = NS; ABA vs. CER *p* = 0.00001; ABA vs. ETA *p* = 0.0025; ABA vs. FIL *p* = 0.00001; ABA vs. SAR *p* = 0.00001; ABA vs. TOC *p* = 0.00001; ABA vs. UPA *p* = 0.00001; ADA vs. BAR *p* = 0.017; ADA vs. CER *p* = 0.0029; ADA vs. ETA *p* = NS; ADA vs. FIL *p* = 0.00001; ADA vs. SAR *p* = 0.00001; ADA vs. TOC *p* = 0.000014; ADA vs. TOF *p* = 0.00001; ADA vs. UPA *p* = 0.00001). BAR does not show differences with CER and ETA. It has a greater percentage of patients in co-treatment compared with FIL *p* = 0.00018 and SAR *p* = 0.004. CER is not different from ETA, TOC, and TOF, but it results in superior outcomes in patients co-treated if compared with FIL *p* = 0.00014 SAR *p* = 0.034 and UPA *p* = 0.00002. ETA has a greater percentage of patients in double therapy compared with FIL (*p* = 0.049), SAR (*p* = 0.02), and TOCI (*p* = 0.0046).

FIL SAR, TOC, TOF, and UPA do not present differences when compared to each other about co-treatments.

Another important feature is the percentage of patients who have failed biologic treatment or tsDMARD therapy before starting the last drug. Comparing the ABA group with other cohorts of treatment, we can see that there are no differences with the BAR, ADA, ETA, SAR, TOC, and TOF groups. There are statistically significant differences with the CER group because the percentage of failure is less than in the ABA group (*p* = 0.0013), and with the FIL group, where the percentage is higher (*p* = 0.044), as well as in the UPA cohort (*p* = 0.044).

About ADA, the only difference is with CER, which has a lower percentage of patients who have failed the previous advanced therapy. The same result is observed for BAR, where the difference is only with CER, which has a lower percentage of patients’ failure (*p* = 0.00093). CER group shows a statistically significant difference with all the other treatments, confirming the lower percentage of patients who have failed biologic or tsDMARD before starting this therapy (CER vs. ETA *p* = 0.042; vs. FIL *p* = 0.0001; vs. SAR *p* = 0.00001; vs. TOC *p* = 0.0006; vs. TOF *p* = 0.0005; vs. UPA *p* = 0.0001). ETA shows differences with FIL *p* = 0.0014, TOFA *p* = 0.009, and UPA *p* = 0.001, having a higher percentage of failure of the previous advanced therapy. FIL has a higher percentage of failure when compared with TOC (*p* = 0.013), UPA has a higher percentage of failure compared with SAR (*p* = 0.045) and TOC (*p* = 0.013). CV risk is a very important condition leading to the choice of the pharmacological treatment; therefore, we have analyzed the percentage of patients with this condition within each treatment group. ABA has a higher percentage of patients with CV risk factors compared with BAR (*p* = 0.00004), CER (*p* = 0.0015), FIL (*p* = 0.005), TOFA (*p* = 0.00028), and UPA (*p* = 0.00001). ADA has a higher percentage compared with BAR (*p* = 0.037), TOF (*p* = 0.00480 and UPA (*p* = 0.00001). The percentage is lower if compared with SAR (*p* = 0.004) and TOC (*p* = 0.006). In the BAR group, the percentage of patients with CV risk factors is lower than in ETA (*p* = 0.037), SAR (*p* = 0.0001), and TOC (*p* = 0.00001), but it results in a higher percentage compared with TOF (*p* = 0.0066). CER has a lower percentage compared with SAR (*p* = 0.00004) and TOC (*p* = 0.00072), and a higher percentage compared with UPA (*p* = 0.002). ETA has a lower percentage compared with SAR (*p* = 0.0047) and TOC (*p* = 0.00038), and a higher percentage compared with TOF (*p* = 0.004) and UPA (*p* = 0.00001). The FIL group shows a lower percentage of patients compared to SAR (*p* = 0.000031) and TOC (*p* = 0.000049), and a higher percentage compared with UPA (*p* = 0.00038). SAR compared to UPA shows a higher percentage of patients with CV risk (*p* = 0.04), as well as TOCI vs. UPA (*p* = 0.00059). No differences in the percentage of patients with hepatotoxicity risk were detected across the different treatment groups. No differences in the risk of TB and other infections were observed among the different treatment groups. High titers of autoantibodies can lead to therapy; therefore, we have analyzed the percentage of patients with this feature in each treatment group. ABA has a higher percentage compared to ADA (*p* = 0.00001), CER (*p* = 0.009), and ETA (*p* = 0.00003). ADA has a low percentage of patients compared to FIL (*p* = 0.00001), CER (*p* = 0.00054), BAR (*p* = 0.00001), SAR (*p* = 0.00006), TOC (*p* = 0.00001), TOF (*p* = 0.00006), and UPA (*p* = 0.00001). BAR has a higher percentage of patients with high titers of autoantibodies compared to CER (*p* = 0.005), ETA (*p* = 0.00001), SAR (*p* = 0.026), and TOF (*p* = 0.026). CER shows a higher percentage compared with ETA (*p* = 0.036) and a lower percentage compared with FIL (*p* = 0.016) and UPA (*p* = 0.02). ETA has a lower percentage compared to FIL (*p* = 0.00005), SARI (*p* = 0.007), TOC (*p* = 0.0018), TOF (*p* = 0.007), and UPA (*p* = 0.00005). No differences among FIL, SAR, TOC, TOF, and UPA are shown. Another important clinical condition impacting the therapy is the presence of interstitial lung disease (ILD), but a statistically significant difference is revealed only between ABA and ETA (*p* = 0.0096). The number of patients with neoplasia is low, and there are no statistically significant differences comparing the different groups of treatment. About the presence of diabetes, there are no statistically significant differences in the groups of treatment. In RA, 34.2% of patients were D2T, 54.8% were in monotherapy (TOC-SAR-UPA-FIL). Only 24.3% of patients had concomitant MTX therapy.

### 3.2. Spondyloarthritis Patients

For each group of arthritis, we have performed the same analysis aimed at identifying the different conditions that characterize the patients treated with different drugs. All the items are reported in Table 3.

About age, there is only one statistically significant difference between CER patients (49 ± 11.48 years old) versus ETA patients (61.08 ± 12.24 years old) (*p* = 0.0176). It is very important to evaluate the percentage of patients treated in combination therapy with biologic drugs and csDMARDs. The statistically significant differences are the following: ADA vs. ETA (*p* = 0.002) and vs. SEC (*p* = 0.00004); in the ADA group, the percentage of patients co-treated is higher than in the other two cohorts of treatments. CER has a higher percentage of patients in combination therapy compared with ETA (*p* = 0.043) and SEC (*p* = 0.00022). In the INF group, the percentage is higher than in the ETA group (*p* = 0.0002), in the GOL group (*p* = 0.044), and in the SEC group. However, in the GOL cohort, the percentage is higher than in the SEC group (*p* = 0.0008). Another important feature is the percentage of patients who have failed biologic treatment or tsDMARD therapy before starting the last drug. ADA has a lower percentage of patients who have failed the biologic therapy than CER (*p* = 0.00027), GOL (*p* = 0.00001), INF (*p* = 0.031), and SEC (*p* = 0.00001). CER shows a higher percentage of failure of biologic therapy compared with ETA (*p* = 0.0049) and INF (*p* = 0.048), but it has a lower percentage when compared with SEC (*p* = 0.026). Both GOL and SEC have a higher percentage compared with ETA (*p* = 0.00013 and *p* = 0.00001). Finally, GOL has a higher percentage compared with INF (*p* = 0.0022), and INF has a lower percentage of failure of biologic therapy than SEC (*p* = 0.00027).

CV risk is a very important condition that leads to pharmacological treatment; therefore, we have analyzed the percentage of patients with this condition within each treatment group. ADA shows a higher percentage of patients with CV risk factors compared with CER (*p* = 0.00001), but it has a lower percentage compared with GOL (*p* = 0.033) and SEC (*p* = 0.0087). CER has a lower percentage compared with ETA (*p* = 0.00016), GOL (*p* = 0.00001), INF (*p* = 0.00001), and SEC (*p* = 0.00001). GOL, INF, and SEC have a higher percentage of patients with CV risk than ETA (*p* = 0.003; *p* = 0.02; *p* = 0.0005, respectively).

About hepatotoxicity, no differences between the different groups of treatment have been found. No one patient shows TB infection and other infection risk factors, except for the SEC group, where 14.3% patients show this risk. Regarding the extra-articular manifestations, no differences between the groups of treatment have been found. If we consider the percentage of patients with enthesitis/dactylitis, statistically significant differences are in the ADA group vs. the SEC group (*p* = 0.00059) because in the SEC group, there is a higher percentage of patients with this manifestation, and in the ETA group vs. the SEC group (*p* = 0.033). No one with neoplasm in the group of patients affected by SpA is present. No statistically significant differences for diabetes are shown. Only 5.82% of patients had concomitant MTX therapy.

### 3.3. Psoriatic Arthritis Patients

As well as for the other two types of arthritis, we have analyzed whether some features can lead to the treatment choice. In Table 4, it is possible to see the characteristics that we have analyzed in the different groups of treatment.

In relation to the age of patients, we can see a statistically significant difference between ADA and IXE group (*p* = 0.036) because the average is higher in IXE cohort; patients treated with APR are older compared to those treated with SEC (*p* = 0.033) and TOF (*p* = 0.045), as well as patients treated with IXE are older than those treated with SEC (*p* = 0.00005) and TOF (*p* = 0.024). In the ADA group, the concomitant treatment is more frequent compared with the CER (*p* = 0.0009) and SEC groups (*p* = 0.029). There is a statistically significant difference between the APR and CER groups, where in the APR group, the percentage of patients treated with other drugs is higher (*p* = 0.0001). BIM shows a higher percentage of patients co-treated compared to ETA (*p* = 0.044), IXE (*p* = 0.014), and SEC (*p* = 0.0016). In the CER group, no patient is treated with other drugs. Therefore, it is possible to identify statistically significant differences with ETA (*p* = 0.0086), IXE (*p* = 0.0035), SEC (*p* = 0.029), and TOF (*p* = 0.0004). TOF has a higher percentage of patients co-treated compared to SEC (*p* = 0.011).

Another important feature is the percentage of patients who have failed bDMARD treatment or tsDMARD therapy before starting the last drug. The group treated with ADA shows a higher percentage of patients who have failed bDMARD or tsDMARD compared with ETA (*p* = 0.0009) and a lower percentage compared to IXE (*p* = 0.007) and TOF (*p* = 0.007).

The presence of CV risk can impact the choice of treatment. BIM and IXE show a higher percentage of patients with CV risk compared with ADA (*p* = 0.0049, *p* = 0.0035). BIM has more patients with CV risk compared with ETA (*p* = 0.011) and SEC (*p* = 0.002). IXE has a higher percentage compared with CER (*p* = 0.049), as well as vs. ETA (*p* = 0.0087) and SEC (*p* = 0.002). TOF shows no patient with risk factors. Therefore, the differences are statistically significant versus all the other drugs (*p* = 0.00001).

No differences between the treatment groups regarding the risk of hepatotoxicity are found, nor are there differences in TB and other infection risks or extra-articular manifestations.

Enthesitis and dactylitis are frequent; hence, we have evaluated the differences in terms of the percentage of patients affected by these manifestations in the different treatment groups. The ADA group has a lower percentage compared to the APR (*p* = 0.0001), BIM (*p* = 0.0001), CERT (*p* = 0.022), IXE (*p* = 0.0001), SEC (*p* = 0.001), and TOF (*p* = 0.0044) groups. APR group has a higher percentage compared with the CER (*p* = 0.038) and ETA (*p* = 0.001) groups, while the IXE group has a higher percentage than the APR one (*p* = 0.044). BIM has a higher percentage compared to CER (*p* = 0.006) and ETA (*p* = 0.0001) groups, while CER has a higher percentage compared with the ETA group (*p* = 0.0036). IXE and SEC groups show more patients with this problem compared to the CER group (*p* = 0.0004 and *p* = 0.0007, respectively). IXE, SEC, and TOF groups have a higher percentage compared to ETA one (*p* = 0.0001, *p* = 0.00001, and *p* = 0.0003, respectively). IXE and SEC groups have a higher percentage compared with the TOF group (*p* = 0.019 and *p* = 0.031, respectively). Regarding neoplasm, the differences are between the APR group, which has 71% of patients with this problem, and the other treatment groups, which have percentages ranging from 0 to 5%, with no statistically significant differences between them. Between APR and the other treatments, the values of “*p*” are the following: vs. ADA *p* = 0.00018, BIM *p* = 0.042, ETA *p* = 0.0002, IXE *p* = 0.0046, and SEC *p* = 0.00045. No differences in the percentage of patients affected by diabetes are detected across the different treatment groups. Only 11.37% of patients had concomitant MTX therapy.

## 4. Discussion

Data from the statistical analysis in our study about a real-life population of patients are correlated with the evidence from the scientific literature. For patients with RA, it emerges that ABA is the most used therapy in the elderly, in agreement with data that place ABA as the b-DMARD with a low infection risk [6]. Data from an international observational study of five registries involving a total of 6450 patients showed a low risk of infection and hospitalization. Adverse reactions (ARs) for opportunistic infections, including tuberculosis, were low. These data were consistent with the known safety profile of ABA [7]. ABA is also the b-DMARD used predominantly in association with MTX as demonstrated by its efficacy data [8]. In our cohort, ABA has been mainly prescribed in patients who present RF and ACPA positivity (96.7%). Data from the NORD-START trials on 778 patients demonstrated that ABA treatment was associated with a better response in the RF and/or ACPA-positive subgroup of patients at 24 weeks [9]. ABA treatment was also chosen in 48% of patients with CV disease and 16% of patients with associated ILD [10,11]. As regards monotherapy, the most used therapies are represented by TOC, SAR (IL-6 inhibitors 41%), and FIL as a Jak inhibitor. The association with MTX was present only in 7–12% of patients, because in many cases, patients presented in their history ineffectiveness or adverse events from MTX [12]. The AMBITION study established TOC as an initial biological agent (monotherapy), demonstrating statistically superior clinical efficacy compared to a standard MTX dose regimen (20 mg/week). While ACR20 was used as a regulatory endpoint, TOC also achieved higher ACR50 and ACR70 response rates that are more clinically relevant measures of meaningful improvement in RA [13]. Data from the GISEA registry on 246 patients demonstrate that FIL survival rate was 84.5% at the 6-month and 75.8% at 12-month follow-up, which were comparable in monotherapy or in combination therapy, irrespective of GC intake [14]. CER is the most widely used anti-TNF drug in the young population, chosen in relation to the desire for pregnancy in these patients [15]. Furthermore, the population with RF positivity was also treated with CER in 54% of cases. Recent research suggests that TNFi lacking the IgG1-Fc fragment, like CER pegol, may offer advantages over TNFi with an IgG1-Fc fragment in RF-positive patients by preventing immune complex formation [16]. As for patients with CV risk present in 44% of our RA case series, together with ABA and IL-6 inhibitors [10,11,12], ADA and ETA are the most used anti-TNFs, with 33% of patients. These data are significant compared to Jak inhibitors, in line with the data from the ORAL-Surveillance study [17]. Among Jak-i, BAR is the least used in patients with CV events due to its known activity on Jak-2 [18], while the choice in the Jak-i class favors FIL, which appears to have a lesser impact on the lipid profile than other molecules [19,20]. The choice of a monotherapy with JAK inhibitors, ABA, or IL-6 inhibitors was mainly due to MTX withdrawal for inefficacy or side effects in 34.3% of patients defined as D2T-RA [21]. Furthermore, monotherapy (TOCI-SARI-UPA-FIL) was present in 54.8% of patients with RA [22,23]. The presence of monotherapy and D2T population influences the low percentage of anti-TNF biosimilars (ETA-ADA) in our case study (24.3%).

The analysis of the population with SpA shows that treatment with anti-TNF biosimilars is the most used in 65.1% of patients. In relation to age, treatment with ETA is the most used in older patients due to its known lower risk of infection and excellent tolerability in the population over 65 years of age [24], while as for RA, CER is the therapy of choice in young people; CER treatment in young pregnant women with SpA has been described in a case series [25]. Compared to anti-IL-17A (SEC), patients treated with anti-TNF biosimilars show a greater association with csDMARD (MTX-sulfasalazine). In particular, INF biosimilar shows the greatest association with MTX (12.5%); this association is due to the known effects of MTX on the reduction in immunogenicity and formation of anti-drug antibodies to INF [26]. ADA is the anti-TNF with the lowest percentage of previous failures, and ETA is the one with the lowest risk of CV events [27]. SEC is the most used in cases of previous risk of tuberculosis and in extra-articular manifestations of enthesitis/dactylitis, followed by ETA [28,29]. Analysis of the population affected by PsA shows treatment with anti-TNF biosimilars in 52.4% of patients. The population treated with anti-IL-17 (IXE, SEC, BIM) represents 29.8%. Patients with the highest concomitant therapy with csDMARD are treated with ADA, where MTX is present in 15.7%; this population is also the one with the highest probability of failure as first therapy. IXE represents the main therapeutic choice in patients with CV risk events compared to anti-TNF and other anti-IL-17 (SEC-BIM) [30]; furthermore, the population treated with IXE has a mean age greater than 65 years [31]; it is also the most followed therapy in the treatment of patients with enthesitis and dactylitis compared to other anti-IL-17, anti-TNF and Jak inhibitors [32]. Finally, the analysis has shown that 71% of APR prescriptions are reserved for patients with previous cancer [33]; for the others, the most important comorbidity was COPD and CV events [34]. The prescription choices based on ITABIO belong to a regional indication of Tuscany and, in particular, of the USL-Toscana Centro, but do not reflect the indications given by the Italian Society of Rheumatology in the prescription of DMARD b-DMARD and tS-DMARD in RA, SpA, and PsA [35,36,37].

## 5. Conclusions

Our retrospective cross-sectional analysis of prescriptions for patients with RA, SpA, and PsA showed how the ITABIO recommendations [4,5] can guide towards correct prescribing appropriateness. We respected the payer indications for SpA and PsA, and our patient population was treated with 65.1% and 52.4% of anti-TNF biosimilars, respectively. The variables represented by enthesitis and dactylitis or cardiovascular risk complications, instead, orient towards treatment with anti-IL-17A antibodies. In the group of patients with RA, the presence of a D2T population in 34.3%, the suspension of MTX, and the presence of monotherapy in 54.8% of patients reduced the percentage of patients treated with anti-TNF biosimilars (24.3%). The limitation of our study, resulting from the database formulated by the payers, is the lack of a clinimetric system that assessed disease activity and the reporting of adverse events that led to the suspension of therapies. Future directions, such as the presence of serum biomarkers or pathophenotype in synovial biopsy, could guide more targeted therapy from the early stages of rheumatoid disease, even if epigenetics presents different expressions at different times of the disease course.

## Figures and Tables

**Table 1:** Distribution of treatments with drugs with multiple indications.

	Ada	Certo	Eta	Gol	Upa	Tof	Apr	Bim	Gus	Inf	Ixe	Sec
RA	6.3	3.9	18	0.7	9.3	2.7	0	0	0	0	0	0
PsA	18	3.8	33	0.47	2.16	2.4	6.6	3.8	1.9	1.4	10.4	15.6
SpA	38	6.8	20.3	7.8	2.5	0	0.8	0.8	1.7	6.8	2.5	12
*p* RA vs. PsA	0.00033	NS	0.004	NS	0.001	NS	0.02	NS	NS	0.021	0.015	NS
*p* RA vs. SpA	0.00001	NS	NS	0.0005	0.03							
*p* PsA vs. SpA	0.003	NS	NS	0.007	NS							

**Table 2:** Characteristics of RA patients.

Drug	Age	Concomitant Therapy	Biol or Ts Failure	CV Risk Factors	Risk of Hepatotoxicity	Risk of TB and Other Infections	FR and/or Anti-CCP	ILD	Neoplasm	Diabetes
abatacept (11%)	71.19 ± 10.65	MTX 74%	64%	48%	3.20%	9.60%	96.70%	16%	3.20%	6.40%
		LEF 19%								
adalimumab (6.3%)	61.83 ± 10.65	MTX 55.5%	72.20%	33.30%	0	0	16.60%	0.00%	0.00%	11%
		LEF 5.5%								
baricitinib (2.3%)	62.5 ± 16.11	MTX 33%	66%	16%	0	16%	100%	16%	0%	0%
certolizumab (3.9%)	50.64 ± 16.47	MTX 18%	27%	18%	9%	18%	54%	0%	0%	0%
		LEF 9%								
etanercept (18%)	66.9 ± 16.23	MTX 38.5%	48%	33%	3.80%	11.50%	30.70%	0.00%	1.90%	5.70%
		LFE 7.7%								
filgotinib (4.8%)	70.5 ± 14.31	MTX 7%	100%	21%	0	0	93%	0%	0%	0%
golimumab (0.7%)	***	***	***	***	***	***	***	***	***	***
sarilumab (11%)	70.22 ± 13.74	MTX 12.5%	65.60%	68.70%	0.00%	6.20%	62.50%	0.00%	3%	9.30%
tocilizumab (30%)	70.5 ± 13.86	MTX 11.6%	58.10%	66.20%	2.30%	4.60%	68.60%	14%	4.60%	11.60%
		LEF 1.1%								
tofacitinib (2.7%)	52.63 ± 12.59	MTX 12.5%	87.50%	12.50%	0	0	62.50%	12%	0	0
upadacitinib (9.3%)	64.9 ± 10.53	MTX 3.7%	100%	3.70%	0	0	92.60%	3.70%	3.70%	0

***: non-significant data.

**Table 3:** Characteristics of the SPA group.

Drug	Age	Concomitant Therapy	Biol or Ts Failure	CV Risk Factor	Risk of Hepatotoxicity	Risk of TB and Other Infections	Extra Articular Manifestations	Enthesitis/Dactylitis	Neoplasm	Diabetes
adalimumab (38%)	57.61 ± 12.88	MTX 6.8%	18%	23%	4.50%	0.00%	41.00%	6.80%	0.00%	6.80%
		LEF 13.6%								
apremilast (0.8)	***	***	***	***	***	***	***	***	***	***
bimekizumab (0.8)	***	***	***	***	***	***	***	***	***	***
certolizumab (6.8%)	49 ± 11.48	MTX 12.5%	62%	0%	0%	0%	25%	25%	0%	13%
etanercept (20.3%)	61.08 ± 12.24	SSZ 4%	29%	17%	4.10%	0.00%	0.00%	16.60%	0.00%	4.10%
golimumab (7.8%)	59.33 ± 11.75	MTX 11%	78%	44%	11%	0%	44%	22%	0%	22%
guselkumab (1.7%)	***	***	***	***	***	***	***	***	***	***
infliximab (6.8%)	53.88 ± 13	MTX 12.5%	37.50%	37.50%	0.00%	0.00%	37.50%	25.00%	0%	12.50%
		SSZ 12.5%								
ixekizumab (2.5%)	***	***	***	***	***	***	***	***	***	***
secukinumab (12%)	60.71 ± 12	0%	100.00%	50.00%	0%	14.30%	0%	71%	0	7%
upadacitinib (2.5%)	***	***	***	***	***	***	***	***	***	***

***: non-significant data.

**Table 4:** Characteristic of the PSA group.

Drug	Age	Concomitant Therapy	Biol or Ts Failure	CV Risk Factors	Risk of Hepatotoxicity	Risk of TB and Other Infections	Extra Articular Manifestations	Enthesitis/Dactylitis	Neoplasm	Diabetes
adalimumab (18%)	60.26 ± 13.29	MTX 15.7%	55%	29%	2.60%	5.20%	5.20%	18.40%	0.00%	10.50%
		LEF 2.6%								
apremilast (6.6%)	65.29 ± 10.11	MTX 14%	78%	43%	0%	7%	7%	64%	71%	14%
bimekizumab (3.8%)	65.88 ± 11.38	MTX 25%	75%	62.50%	0%	0%	0%	75%	0%	0%
certolizumab (3.8%)	53 ± 19.10	0%	63%	38%	0%	0%	0%	38%	0%	0%
etanercept (33%)	62.77 ± 13.57	MTX 8.7%	17.30%	32%	4.30%	4.30%	0%	10%	1%	10%
		LEF 1.4%								
		SSZ 1.4%								
filgotinib (0.47%)	***	***	***	***	***	***	***	***	***	***
golimumab (0.47%)	***	***	***	***	***	***	***	***	***	***
guselkumab (1.9%)	***	***	***	***	***	***	***	***	***	***
infliximab (1.4%)	***	***	***	***	***	***	***	***	***	***
ixekizumab (10.4%)	67.41 ± 10.33	MTX 9%	100.00%	63.60%	0%	4.50%	0%	100%	5%	0%
secukinumab (15.6%)	56.67 ± 11.49	MTX 6%	75.70%	27.20%	3%	6%	0%	97.00%	0%	6%
tofacitinib (2.4%)	53.8 ± 10.06	MTX 20%	100.00%	0%	0%	0%	0%	60%	0%	0%
upadacitinib (2.16%)	***	***	***	***	***	***	***	***	***	***

***: non-significant data.

## Data Availability

The raw data supporting the conclusions of this article will be available from the authors without undue reservation.
